# Lifetime impact of achondroplasia study in Europe (LIAISE): findings from a multinational observational study

**DOI:** 10.1186/s13023-023-02652-2

**Published:** 2023-03-15

**Authors:** Mohamad Maghnie, Oliver Semler, Encarna Guillen-Navarro, Angelo Selicorni, Karen E. Heath, Gabriele Haeusler, Lars Hagenäs, Andrea Merker, Antonio Leiva-Gea, Vanesa López González, Adalbert Raimann, Mirko Rehberg, Fernando Santos-Simarro, Diana-Alexandra Ertl, Pernille Axél Gregersen, Roberta Onesimo, Erik Landfeldt, James Jarrett, Jennifer Quinn, Richard Rowell, Jeanne Pimenta, Shelda Cohen, Thomas Butt, Renée Shediac, Swati Mukherjee, Klaus Mohnike

**Affiliations:** 1 Department of Pediatrics, IRCCS Istituto Giannina Gaslini, Genoa, Italy; 2 Department of Neuroscience, Rehabilitation, Ophthalmology, Genetics, Maternal and Child Health, University of Genova, Genoa, Italy; 3 ERN-BOND, Dublin, Ireland; 4 Faculty of Medicine and University Hospital Cologne, Department of Pediatrics, University of Cologne, Cologne, Germany; 5 Sección de Genética Médica, Servicio de Pediatría, Hospital Clinico Universitario Virgen de la Arrixaca, Murcia, Spain; 6 CIBERER, ISCIII, Madrid, Spain; 7 UOC Pediatria, Como, Italy; 8 Hospital Universitario la Paz, Institute of Medical and Molecular Genetics and Skeletal Dysplasia Multidisciplinary Unit (UMDE), Madrid, Spain; 9 Vienna Bone and Growth Center, Department of Pediatrics and Adolescent Medicine, Medical University of Vienna, Vienna, Austria; 10 Karolinska University Hospital, Stockholm, Sweden; 11 Department of Women’s and Children’s Health, Karolinska Institutet, Stockholm, Sweden; 12 Instituto de Investigación Biomédica de Málaga-IBIMA, Hospital Universitario Virgen de la Victoria, Málaga, Spain; 13 Klinisk Genetisk Afdeling and Centre for Rare Diseases, Aarhus University Hospital, Aarhus, Denmark; 14 Rare Disease Unit, Fondazione Policlinico Universitario Agostino Gemelli IRCCS, Rome, Italy; 15 IQVIA, Stockholm, Sweden; 16 BioMarin (UK) Limited, London, UK; 17 BioMarin Pharmaceutical Inc., Novato, CA USA; 18 Otto-Von-Guericke Universität, Universitätskinderklinik Magdeburg, Magdeburg, Germany

**Keywords:** Achondroplasia, Skeletal dysplasia, Fibroblast growth factor receptor 3 (FGFR3), Natural history, Disease burden

## Abstract

**Background:**

Achondroplasia, caused by a pathogenic variant in the fibroblast growth factor receptor 3 gene, is the most common skeletal dysplasia. The Lifetime Impact of Achondroplasia Study in Europe (LIAISE; NCT03449368) aimed to quantify the burden of achondroplasia among individuals across a broad range of ages, including adults.

**Methods:**

Demographic, clinical and healthcare resource use data were collected from medical records of achondroplasia patients enrolled in 13 sites across six European countries in this retrospective, observational study. Descriptive statistics or event rates per 100 person-years were calculated and compared across age groups as well as by history of limb lengthening. Patient-reported outcomes (quality of life [QoL], pain, functional independence, work productivity and activity impairments) were evaluated using questionnaires at the time of enrolment. An exploratory analysis investigated correlations between height (z-score or centimetres) and patient-reported outcomes.

**Results:**

Overall, 186 study patients were included, with a mean age of 21.7 ± 17.3 years (range 5.0–84.4). At least one complication or surgery was reported for 94.6% and 72.0% of patients, respectively, at a rate of 66.6 and 21.5 events per 100 person-years. Diverse medical and surgical complications were reported for all ages in a bimodal distribution, occurring more frequently in the youngest and oldest age groups. A total of 40 patients had previously undergone limb lengthening (capped at 20% per the study protocol). The most frequent surgery types varied by age, in line with complication profiles. Healthcare resource use was high across all age groups, especially among the youngest and oldest individuals, and did not differ substantially according to history of limb lengthening. Compared to general population values, patients reported impaired QoL particularly for physical functioning domains. In addition, patients reported difficulty carrying out daily activities independently and pain starting in childhood. Patient height correlated with multiple patient-reported outcomes.

**Conclusions:**

The findings of this study suggest that, across an individual’s lifetime, achondroplasia is associated with multisystem complications, reduced QoL and functionality, and increased pain. These results highlight the large amount of healthcare resources that individuals with achondroplasia require throughout their lifespans and provide novel insights into current achondroplasia management practices across Europe.

*Trial registration* ClinicalTrials.gov, NCT03449368, Submitted 14 December 2017 – prospectively registered, https://clinicaltrials.gov/ct2/show/record/NCT03449368

**Supplementary Information:**

The online version contains supplementary material available at 10.1186/s13023-023-02652-2.

## Introduction

Achondroplasia is the most common skeletal dysplasia, with an estimated prevalence of approximately 1:22,000 live births and over 250,000 people worldwide affected [1, 2]. In Europe, the pooled prevalence of achondroplasia is 3.5 per 100,000 live births [1]. The condition is caused by a heterozygous pathogenic variant in the fibroblast growth factor receptor 3 gene (*FGFR3*), resulting in impaired endochondral bone growth [3].

As a result of their condition, individuals with achondroplasia present with a range of clinical features, the most recognisable of which is extreme short stature; on average, adult patients of both sexes have a height that is 6.0 standard deviation scores [SDS] below the mean for unaffected individuals [4]. Other clinical features include disproportionately short limbs, macrocephaly, a small chest and midfacial retrusion [3, 4]. In addition, individuals typically experience a range of serious medical and surgical complications throughout their lifetimes including neurological, orthopaedic, cardiorespiratory, ear, nose and throat (ENT) and dental problems [3, 5]. Foramen magnum stenosis is often present at birth and, in the absence of timely surgical intervention, can result in neurologic complications and even sudden death in infancy as a result of brainstem compression [3]. There is a need to further elucidate how complications are experienced across the lifespan of individuals with achondroplasia. Further, while a recent natural history study indicates that individuals with achondroplasia in the US experience increased rates of surgical procedures, [6] it would be valuable to understand how surgeries and other healthcare resource needs are experienced across the lifespan of individuals with achondroplasia.

Alongside physical complications, studies indicate that individuals with achondroplasia experience reduced quality of life (QoL) and functionality compared with unaffected individuals as well as low self-esteem, depression and anxiety [7–9]. However, there is a need to understand these patient-reported outcomes among larger and more geographically- and age-diverse achondroplasia populations using a broader range of questionnaires. Further, while height (SDS and/or in cm) outcomes have been used as proxies for QoL in studies of achondroplasia [7, 10, 11]. there is a need to investigate the relationship between these variables in larger achondroplasia populations [10–12].

The Lifetime Impact of Achondroplasia Study in Europe (LIAISE; NCT03449368) was designed to quantify the impact of achondroplasia across the age spectrum within Europe by assessing the clinical and surgical burden, healthcare resource use and QoL of affected individuals. Here, the results of LIAISE are presented, in addition to the findings of an exploratory analysis between the height outcomes and patient-reported outcomes of the study population.

## Methods

LIAISE was a multinational, retrospective, observational natural history study conducted across six countries in Europe to investigate the lifetime disease burden of achondroplasia. The study also included a cross-sectional component to assess QoL.

### Sample population

Patients with achondroplasia were recruited from December 2017 to February 2020. Recruitment took place at 13 sites across Austria, Denmark, Germany, Italy, Spain and Sweden. Patients were identified for enrolment via one of three routes: (1) during routine hospital visits; (2) from lists of former patients; or (3) through achondroplasia patient organizations, other achondroplasia-related forums (including social media sites), or other healthcare professionals.

Patients were required to be ≥ 5 years old at the time of enrolment, with genetically and/or clinically and/or radiologically confirmed diagnosis of achondroplasia and at least five years of documented medical history data. Patients who were participating or had participated in a clinical trial (or a non-clinical or low-interventional study) within six months prior to enrolment were excluded from the study. The number of patients included with previous limb lengthening surgery was capped at 20% to minimise selection bias.

All included patients (or the parents or legally accepted representatives thereof) provided informed written consent and were considered to have the cognitive and linguistic capacities necessary to understand and take part in the study. The study was conducted in compliance with the Declaration of Helsinki and all sites received independent ethics committee approval.

### Data collection

The data underlying this study were extracted from patients’ medical records or collected directly from patients using patient-reported outcomes (see below). Data extraction was performed by the site/study physician, coordinator or nurse. At some sites, data extraction was performed by an independent data abstractor who visited the centre. The extracted data was collated into a pre-defined electronic case report form. Data collection from medical records was supplemented by data provided by the patient regarding their own medical history and, if necessary, confirmed by an additional healthcare provider.

Specific data categories collected using patient medical records included: general demographics (age, sex), details of achondroplasia diagnosis (method, type of variant), height measurements, medical history (Medical Dictionary for Regulatory Activities [MedDRA] coded complications and surgical procedures of pre-defined categories) and use of healthcare resources prior to enrolment (medication, visits to/examinations by healthcare professionals, examinations to inform medical or surgical management, hospitalisations). A full list of data categories collected using patient medical records is provided in Additional File 1.

Prior to analysis, general medical history data were mapped to one of ten pre-defined complications of interest (list presented in Additional File 2). These included disease-related complications which were commonly reported in the literature or known to have substantial impact upon individuals with achondroplasia.

Patient-reported outcome data relating to QoL, physical and psychosocial function, pain, emotional or coping status and activity impairments of patients were collected using a booklet of validated, structured questionnaires provided to patients as part of their routine hospital visits (or sent by post if the patient was not attending a hospital). Questionnaires for patients aged 5–17 years were the Quality of Life in Short Stature Youth (QoLISSY) questionnaire , [13] the Pediatric Quality of Life Inventory (PedsQL), [14] the paediatric Functional Independence Measure (WeeFIM) [15] and the Adolescent Pediatric Pain Tool (APPT) [16]. Questionnaires for patients ≥ 18 years old included the Work Productivity and Activity Impairment Questionnaire for a Specific Health Problem (WPAI:SHP; Version 2), [17] the EQ-5D-5L questionnaire, [18] the Brief Pain Inventory Short Form (BPI-SF) [19] and the Nottingham Health Profile (NHP) questionnaire [20]. A summary of characteristics for each questionnaire, including their targeted population and a description of their outcomes, are presented in Additional File 3.

### Statistical analysis

Data were stratified by patient age (5–10, 11–15, 16–20, 21–30, 31–40, 41–50, 51–60, > 60 years). Demographic and clinical characteristics were summarised using proportions, medians, means, SDs, and ranges; no formal hypothesis testing was conducted. To account for the varying durations of available medical history, event rates per 100 person-years (for complications, surgical procedures, and healthcare resource use) were calculated using the total number of events divided by the total number of patient historical years and multiplied by 100. The start of the historical follow-up period for each patient was estimated using the earliest medical event recorded in the case report form. All patient-reported outcomes were scored according to their manuals and compared with general population reference data, if available. All analyses were stratified by age and history of limb lengthening. No imputation was planned for missing values, with the exception of dates and results of the BPI-SF, QoLISSY and PedsQL questionnaires if scores could not be calculated. Further information on imputation of missing data can be found in Additional File 4. Height-for-age curves of the LIAISE study population (by sex and excluding patients who had undergone limb lengthening) were plotted against published achondroplasia growth data [21].

An exploratory analysis was conducted to investigate the correlation between height z-score (SDS) or absolute height (centimetres [cm]) and patient-reported outcomes (QoL, pain and functionality). These analyses were carried out only for the sub-set of patients who had height measurements within one year of enrolment as well as sufficient patient-reported outcome data (see Additional File 5 for details of analysis methods). Associations between height z-score or absolute height and QoL outcomes were investigated using ordinary least square (OLS) regression. In addition to height or height z-score, independent variables in the models included age, gender and complications. The choice to include z-score or height was based on qualitative assessment of the strength and significance of the estimated correlation coefficients between the two variables and each modelled outcome, respectively. For both correlation and regression analyses, height z-scores (age- and gender-matched) were computed using World Health Organisation growth tables by gender [22]. Of note, all p-values and any statistical significance should be considered exploratory.

## Results

### Patient characteristics

The baseline characteristics of the study sample are presented in Table 1. Overall, 186 patients with achondroplasia were eligible for inclusion, providing a total of 2,384.6 years of historical medical data. The median duration of historical follow-up per patient was 9.4 years (min, max: 2.0, 55.0). Though there was a broad age range at enrolment (5.0 to 84.4 years), the majority of patients were children (< 16) when they enrolled in the study. Female patients formed a slightly greater proportion of the total study population than males (54.3% vs 45.7%, respectively). In total, 21.5% of patients had undergone limb lengthening prior to enrolment (Table 2), which slightly exceeded the intended cap of 20%. These patients were predominantly (75%) female and had a slightly higher mean age at enrolment than those who had not undergone this procedure (24.2 years vs 21.0 years). Patients who had undergone limb lengthening surgery were primarily from Italy and Spain, where limb lengthening surgery is practiced more frequently as per standard of care [23]. Additional File 6 presents the height-for-age curves of the LIAISE study population (by sex and excluding patients who had undergone limb lengthening), plotted against published achondroplasia growth data [21].

**Table 1:** Baseline characteristics of the study population

Baseline characteristic	Study population (*N* = 186)
Historical data collected (years)*	
Total	2384.6
Median (min, max)	9.4 (2.0, 55.0)
*Country of enrolment, n (%)*	
Denmark	10 (5.4)
Germany	64 (34.4)
Sweden	9 (4.8)
Austria	3 (1.6)
Italy	57 (30.6)
Spain	43 (23.1)
*Age at time of enrolment (years), n* (%)	
Mean (SD)	21.7 (17.3)
Median (Q1,Q3)	14.9 (8.9, 30.8)
Min, Max	5.0, 84.4
*Age subgroups at enrolment (years), n *(%)	
5–10	66 (35.5%)
11–15	36 (19.4%)
16–20	17 (9.1%)
21–30	22 (11.8%)
31–40	14 (7.5%)
41–50	15 (8.1%)
51–60	8 (4.3%)
> 60	8 (4.3%)
*Gender, n* (%)	
Male	85 (45.7%)
Female	101 (54.3%)
*Age at diagnosis of achondroplasia (months), n* (%)	
N	137
Median (Q1, Q3)	0.0 (0.0, 6.0)
10th percentile	0.0
90th percentile	35.0
*Type of confirmation of diagnosis of achondroplasia, n *(%)	
Confirmed first by a specialist through medical examination and/or radiological assessment followed by genetic testing	80 (43.0%)
Confirmed by medical examination and/or radiological assessment with no genetic testing	69 (37.1%)
Confirmed first by genetic testing followed by a specialist through medical examination and/or radiological assessment	28 (15.1%)
Confirmed by genetic testing with no confirmation by a specialist through medical examination and radiological assessment	9 (4.8%)
*FGFR3* variant**, n (%)	
N	99
c.1138G > A p.Gly380Arg	78 (78.8%)
c.1138G > C p.Gly380Arg	3 (3.0%)
Other***	18 (18.2%)

^*^Five patients had less than five years of recorded medical history data; however, the study investigator(s) overseeing these patients confirmed that five years of medical data had been reviewed and so confirmed their eligibility for inclusion

^**^106 patients provided consent for the collection of genetic test information; 102 had the presence of a pathogenic variant in FGFR3 confirmed (data were missing for the remaining 4 patients). Of these, 99 had the specific type of mutation on the FGFR3 gene confirmed (data were missing for the remaining 3 patients)

^***^15 of these patients had either 1138G > A, 1138G > C or G380R documented, 1 patient had C1620G documented, and test reports were not available for 1 patient. All of these mutations had been coded as during data capture

*FGFR3* Fibroblast growth factor 3, *max* Maximum, *min* Minimum, *Q1* First quartile, *Q3* Third quartile, *SD* Standard deviation

**Table 2:** Demographic and baseline characteristics of the study population by limb lengthening

	Subgroup	
	LL: Yes (*N* = 40)	LL: No (*N* = 146)
*Country of enrolment, n* (%)		
Denmark	1 (2.5)	9 (6.2)
Germany	6 (15.0)	58 (39.7)
Sweden	0	9 (6.2)
Austria	0	3 (2.1)
Italy	19 (47.5)	38 (26.0)
Spain	14 (35.0)	29 (19.9)
*Age, n* (%)		
Mean (SD)	24.2 (12.6)	21.0 (18.4)
Median (Q1, Q3)	18.5 (14.7, 36.8)	13.1 (8.0, 26.8)
Min, Max	7.4, 51.4	5.0, 84.4
*Age subgroups at enrolment (years), n* (%)		
5–10	4 (10.0%)	62 (42.5%)
11–15	10 (25.0%)	26 (17.8%)
16–20	8 (20.0%)	9 (6.2%)
21–30	5 (12.5%)	17 (11.6%)
31–40	7 (17.5%)	7 (4.8%)
41–50	5 (12.5%)	10 (6.8%)
51–60	1 (2.5%)	7 (4.8%)
> 60	0 (0.0%)	8 (5.5%)
*Gender, n* (%)		
Male	10 (25.0%)	75 (51.4%)
Female	30 (75.0%)	71 (48.6%)
*Time Since LL (years)*		
N	36	–
Missing	4	–
Mean (SD)	8.4 (9.7)	–
Median (Q1, Q3)	3.5 (1.0, 16.0)	–
Min, Max	0.0, 28.0	–

*LL* Limb lengthening, *max* Maximum, *min* Minimum, *Q1* First quartile, *Q3* Third quartile, *SD* Standard deviation

The median age at diagnosis was 0.0 years (IQR: 0.0–6.0 months; 10th percentile: 0.0; 90th percentile: 35.0 months). The majority (43.0%) of patients were diagnosed via medical examination and/or radiological assessment and a genetic test; of the 99 who consented to share their genetic results, the most common variant was c.1138G > A; p.Gly380Arg (79%).

### Medical and surgical complications

In total, 94.6% of patients reported a total of 1,587 medical and surgical complications, equating to 66.6 events per 100 patient-years. By broad MedDRA system organ class, the most common complications were disorders with musculoskeletal and connective tissue involvement (12.0 per 100 person-years, 58.6%), nervous system disorders (8.7 per 100 person-years, 61.3%) and infections and infestations (6.2 per 100 person-years, 25.8%). Of the specific complications of interest, events related to ENT issues excluding otitis media were the most common (61.3%) and occurred at a rate of 13.4 events per 100 person-years. Spinal deformities and foramen magnum stenosis were documented in 28.0% of patients at a rate of 3.4 and 2.4 events per 100 person-years, respectively.

**Fig. 1:**
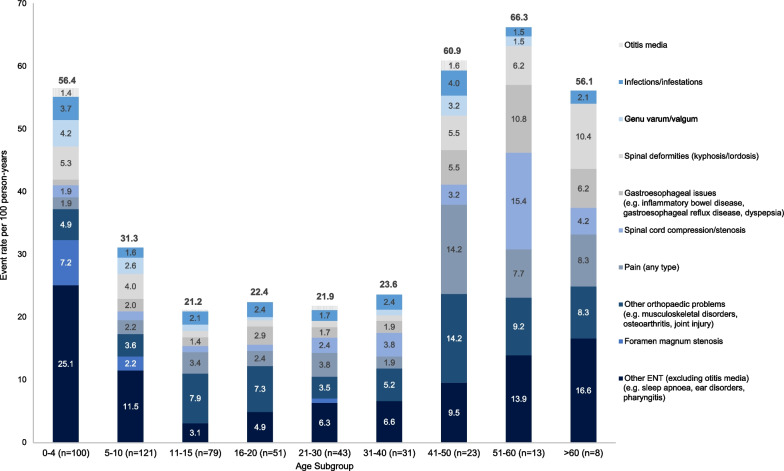
Burden of medical and surgical complications by age at time of event. Each patient may have several complications reported across the lifespan and may therefore be included in multiple age subgroups. Event rates per 100 person-years were calculated as the number of events divided by the total number of patient historical years, multiplied by 100. *ENT* Ear, nose and throat

The distribution of rates and types of specific complications of interest across age groups is presented in Fig. 1. The highest rates of any complication were reported among patients aged < 10 years and > 40 years. While foramen magnum stenosis and ENT issues (excluding otitis media) were reported at the greatest rates between the ages of 0–4 years, the rate of gastroesophageal issues, spinal cord compression and pain increased with age. Spinal deformities (kyphosis/lordosis) followed a U-shaped distribution, occurring at the highest rates among the youngest (0–4 years) and oldest (≥ 40 years) age groups.

Medical history results by limb lengthening subgroup are presented in Table 3. Overall, medical and surgical complications occurred at a rate of 25.6 events per 100-person years among patients with limb lengthening (*N* = 40) and 44.9 events per 100 person-years for those without limb lengthening (*N* = 146). The proportion of patients with at least one medical and surgical complication were similar between the two groups (limb lengthening: 87.5%; no limb lengthening: 87.7%). Other than pain, patients without limb lengthening had numerically higher event rates than those with limb lengthening across all complications of interest. The highest event rate for both groups was reported for other ENT issues, excluding otitis media (limb lengthening: 8.7 events per 100 person-years; no limb lengthening: 15.2 events per 100 person-years).

**Table 3:** Medical history by limb lengthening subgroup

Medical history* *n* (%)/events (rate per 100 person-years)	Subgroup		Overall
	Limb lengthening (*N* = 40)	No limb lengthening (*N* = 146)	*N* = 186
Patient historical years	663.0	1,721.6	2,384.6
Medical and surgical complications**			
≥ 1 general medical history	35 (87.5%)/170 (25.6)	128 (87.7%)/773 (44.9)	163 (87.6%)/943 (39.5)
Foramen magnum stenosis	2 (5.0%)/2 (0.3)	50 (34.2%)/56 (3.3)	52 (28.0%)/58 (2.4)
Gastroesophageal issues	8 (20.0%)/8 (1.2)	24 (16.4%)/48 (2.8)	32 (17.2%)/56 (2.3)
Genu varum/valgum	3 (7.5%)/3 (0.5)	37 (25.3%)/44 (2.6)	40 (21.5%)/47 (2.0)
Infections/infestations	4 (10.0%)/8 (1.2)	23 (15.8%)/45 (2.6)	27 (14.5%)/53 (2.2)
Other ENT issues (excluding otitis media)	24 (60.0%)/58 (8.7)	90 (61.6%)/261 (15.2)	114 (61.3%)/319 (13.4)
Other orthopaedic problems	22 (55.0%)/44 (6.6)	47 (32.2%)/119 (6.9)	69 (37.1%)/163 (6.8)
Otitis media	1 (2.5%)/1 (0.2)	11 (7.5%)/16 (0.9)	12 (6.5%)/17 (0.7)
Pain (any type)	11 (27.5%)/25 (3.8)	34 (23.3%)/66 (3.8)	45 (24.2%)/91 (3.8)
Spinal cord compression/stenosis	10 (25.0%)/10 (1.5)	37 (25.3%)/49 (2.8)	47 (25.3%)/59 (2.5)
Spinal deformities (kyphosis/lordosis)	7 (17.5%)/11 (1.7)	45 (30.8%)/69 (4.0)	52 (28.0%)/80 (3.4)
Surgical burdenb			
≥ 1 surgery/number of surgeries	40 (100%)/223 (33.6)	94 (64.4%)/290 (6.8)	134 (72.0%)/513 (21.5)
Brainstem decompression	2 (5.0%)/2 (0.3)	24 (16.4%)/24 (1.4)	26 (14.0%)/26 (1.1)
Limb lengthening	40 (100%)/113 (17.0)	0	40 (21.5%)/113 (4.7)
Middle ear procedures	6 (15.0%)/8 (1.3)	37 (25.4%)/59 (3.3)	43 (23.1%)/67 (2.8)
Nerve decompression	1 (2.5%)/1 (0.2)	6 (4.1%)/7 (0.4)	7 (3.8%)/8 (0.3)
Shunt insertion	2 (5.0%)/2 (0.3)	5 (3.4%)/5 (0.3)	7 (3.8%)/7 (0.3)
Shunt revision	0	4 (2.7%)/8 (0.5)	4 (2.2%)/8 (0.3)
Spinal correction	2 (5.0%)/4 (0.6)	8 (5.5%)/11 (0.6)	10 (5.4%)/15 (0.6)
Tonsillectomy/adenoidectomy	10 (25.0%)/13 (2.0)	39 (26.7%)/55 (3.2)	49 (26.3%)/68 (2.9)
Other	24 (60.0%)/80 (12.1)	58 (39.7%)/121 (7.0)	82 (44.1%)/201 (8.4)
Healthcare professional visits			
≥ 1 health care professional visit/number of visits	16 (40.0%)/55 (8.3)	97 (66.4%)/460 (26.7)	113 (60.8%)/515 (21.6)
Cardiologist	0	4 (2.7%)/6 (0.3)	4 (2.2%)/6 (0.3)
Dentist	1 (2.5%)/1 (0.2)	2 (1.4%)/2 (0.1)	3 (1.6%)/3 (0.1)
Dermatologist	1 (2.5%)/1 (0.2)	3 (2.1%)/4 (0.2)	4 (2.2%)/5 (0.2)
Dietician	0	4 (2.7%)/4 (0.2)	4 (2.2%)/4 (0.2)
ENT physician and/or surgeon	4 (10.0%)/5 (0.8)	24 (16.4%)/40 (2.3)	28 (15.1%)/45 (1.9)
Emergency Doctor	0	5 (3.4%)/9 (0.5)	5 (2.7%)/9 (0.4)
Endocrinologist	0	3 (2.1%)/7 (0.4)	3 (1.6%)/7 (0.3)
Gastroenterologist	0	3 (2.1%)/5 (0.3)	3 (1.6%)/5 (0.2)
General practitioner	4 (10.0%)/16 (2.4)	23 (15.8%)/120 (7.0)	27 (14.5%)/136 (5.7)
Geneticist	0	5 (3.4%)/7 (0.4)	5 (2.7%)/7 (0.3)
Gynaecologist	0	3 (2.1%)/4 (0.2)	3 (1.6%)/4 (0.2)
Mental Health	1 (2.5%)/1 (0.2)	3 (2.1%)/3 (0.2)	4 (2.2%)/4 (0.2)
Neurologist	1 (2.5%)/1 (0.2)	8 (5.5%)/9 (0.5)	9 (4.8%)/10 (0.4)
Neuropediatric	1 (2.5%)/1 (0.2)	2 (1.4%)/12 (0.7)	3 (1.6%)/13 (0.5)
Neurosurgeon	0	5 (3.4%)/6 (0.3)	5 (2.7%)/6 (0.3)
Occupational therapist	1 (2.5%)/1 (0.2)	5 (3.4%)/5 (0.3)	6 (3.2%)/6 (0.3)
Ophthalmologist	2 (5.0%)/2 (0.3)	7 (4.8%)/7 (0.4)	9 (4.8%)/9 (0.4)
Orthopaedic	3 (7.5%)/6 (0.9)	21 (14.4%)/30 (1.7)	24 (12.9%)/36 (1.5)
Paediatrician	1 (2.5%)/1 (0.2)	7 (4.8%)/56 (3.3)	8 (4.3%)/57 (2.4)
Physiotherapist	7 (17.5%)/10 (1.5)	52 (35.6%)/75 (4.4)	59 (31.7%)/85 (3.6)
Respiratory physician	2 (5.0%)/3 (0.5)	4 (2.7%)/5 (0.3)	6 (3.2%)/8 (0.3)
Speech therapist	4 (10.0%)/4 (0.6)	21 (14.4%)/27 (1.6)	25 (13.4%)/31 (1.3)
Other	2 (5.0%)/2 (0.3)	14 (9.6%)/17 (1.0)	16 (8.6%)/19 (0.8)
Medical or surgical examination			
≥ 1 medical or surgical examination/number of examinations	40 (100%)/821 (123.8)	145 (99.3%)/2,491(144.7)	185 (99.5%)/3,312 (138.9)
Audiology	2 (5.0%)/3 (0.5)	37 (25.3%)/82 (4.8)	39 (21.0%)/85 (3.6)
Cardiovascular	6 (15.0%)/21 (3.2)	36 (24.7%)/75 (4.4)	42 (22.6%)/96 (4.0)
ENT	12 (30.0%)/31 (4.7)	69 (47.3%)/323 (18.8)	81 (43.5%)/354 (14.8)
Gastrointestinal	5 (12.5%)/12 (1.8)	13 (8.9%)/30 (1.7)	18 (9.7%)/42 (1.8)
General surgical	5 (12.5%)/24 (3.6)	7 (4.8%)/17 (1.0)	12 (6.5%)/41 (1.7)
Neurological	12 (30.0%)/46 (6.9)	61 (41.8%)/257 (14.9)	73 (39.2%)/303 (12.7)
Orthopaedic	23 (57.5%)/247 (37.3)	78 (53.4%)/366 (21.3)	101 (54.3%)/613 (25.7)
Radiological	32 (80.0%)/218 (32.9)	98 (67.1%)/519 (30.1)	130 (69.9%)/737 (30.9)
Respiratory	7 (17.5%)/10 (1.5)	31 (21.1%)/79 (4.6)	38 (20.4%)/89 (3.7)
Urological	1 (2.5%)/1 (0.2)	10 (6.8%)/19 (1.1)	11 (5.9%)/20 (0.8)
Other	24 (60.0%)/208 (31.4)	108 (74.0%)/724 (42.1)	132 (71.0%)/932 (39.1)

^*^Each patient may have had several medical history events. Percentages are based on the overall number of subjects in the Full Analysis Set. Event rates per 100 person-years are calculated as the number of events divided by the total number of patient historical years multiplied by 100. Patient historical years are defined as the time between earliest collected history and enrolment date

^**^Terms are coded using MedDRA dictionary and subsequently mapped into 1 of 10 predefined categories described in the Health Economic Exploratory Analysis Plan

*ENT* Ear, nose, throat

### Surgical burden

Overall, 72.0% of patients had undergone at least one surgical procedure. A total of 513 procedures were recorded, at an event rate of 21.5 per 100 person-years. The most common types of surgeries were middle ear procedures (7.0 per 100 person-years; 28.0%) tonsillectomies/adenoidectomies (2.9 per 100 person-years, 26.3%), and brainstem decompression surgeries (1.1 events per 100 person-years, 14.0%).

**Fig. 2:**
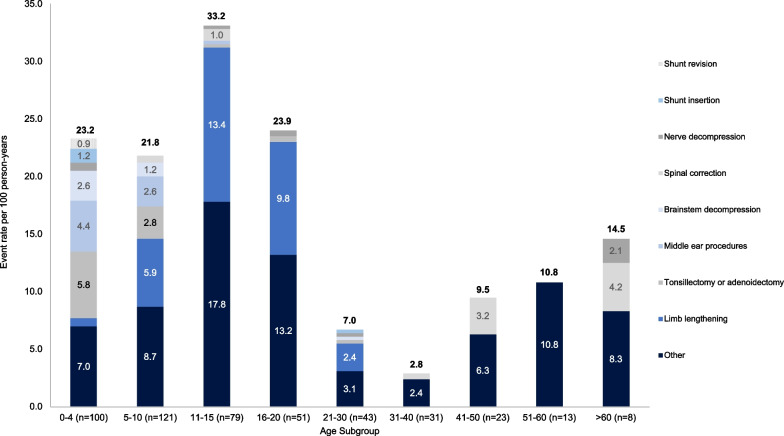
Surgical burden by age at time of event. Each patient may have had several surgeries reported across their lifespan and may therefore be included in multiple age subgroups. Event rates per 100 person-years were calculated as the number of events divided by the total number of patient historical years multiplied by 100. ‘Other’ surgeries were those not pre-defined in the CRF used to record data, and mainly consisted of other orthopaedic procedures (e.g., medical device removal, osteotomy, epiphysiodesis)

The distribution of types of surgical procedure varied by age group (Fig. 2). While tonsillectomies/adenoidectomies, middle ear procedures and brainstem decompressions were predominantly performed when patients were at younger ages (0–10 years), limb lengthening procedures were most frequently performed between the ages of 11–20 years. From ≥ 21 years, ‘other’ types of surgery were the most common, many of which related to other orthopaedic procedures (such as osteotomies) and procedures for the removal of medical devices.

The event rate of surgical procedures overall was 33.6 events per 100 person-years for patients with limb lengthening and 16.8 events per 100 person-years for those without (100% and 64.4% of patients, respectively; Table 3).

### Healthcare resource use

#### Prior medication use

Any medication use prior to enrolment was reported in 72.0% (134/186) of patients, at a rate of 48.4 events per 100 person-years. The most common Anatomical Therapeutic Chemical (ATC) classes were analgesics (8.6 per 100 person-years; 33.3%), antibacterials for systemic use (7.3 per 100 person-years; 37.1%), and anti-inflammatory and anti-rheumatic products (4.7 per 100 person-years; 28.5%).

The total medication frequency varied by age (Table 4). When considered at ATC level 1, anti-infectives for systemic use were only reported by patients up to the age of 30 years, while medications for cardiovascular complications were reported only above the age of 41 years (Table 4).

**Table 4:** Medication use six months prior to baseline by age at enrolment

	Age group, years							
	5–10 (*N* = 66)	11–15 (*N* = 36)	16–20 (*N* = 17)	21–30 (*N* = 22)	31–40 (*N* = 14)	41–50 (*N* = 15)	51–60 (*N* = 8)	≥ 61 (*N* = 8)
*Total medication count per patient*								
n	66	36	17	22	14	15	8	8
Mean (SD)	0.5 (1.42)	0.3 (0.73)	0.6 (0.93)	0.5 (1.41)	0.2 (0.43)	1.0 (1.60)	1.3 (1.28)	1.3 (2.05)
Median	0.0	0.0	0.0	0.0	0.0	0.0	1.0	0.5
Min, Max	0, 8	0, 3	0, 3	0, 6	0, 1	0, 5	0, 3	0, 6
*Total medication frequency, n* (%)								
0	52 (78.8%)	31 (86.1%)	10 (58.8%)	17 (77.3%)	11 (78.6%)	9 (60.0%)	3 (37.5%)	4 (50.0%)
1	5 (7.6%)	3 (8.3%)	4 (23.5%)	3 (13.6%)	3 (21.4%)	2 (13.3%)	2 (25.0%)	2 (25.0%)
2	5 (7.6%)	0	2 (11.8%)	0	0	2 (13.3%)	1 (12.5%)	1 (12.5%)
3	1 (1.5%)	2 (5.6%)	1 (5.9%)	1 (4.5%)	0	0	2 (25.0%)	0
≥ 4	3 (4.5%)	0	0	1 (4.5%)	0	2 (13.3%)	0	1 (12.5%)
*ATC Level 1 Term, n* (%)								
Alimentary tract and metabolism	5 (7.6%)	1 (2.8%)	0	1 (4.5%)	0	2 (13.3%)	1 (12.5%)	1 (12.5%)
Antiinfectives for systemic use	6 (9.1%)	1 (2.8%)	3 (17.6%)	1 (4.5%)	0	0	0	0
Blood and blood forming organs	0	1 (2.8%)	2 (11.8%)	0	2 (14.3%)	0	0	0
Cardiovascular system	0	0	0	0	0	1 (6.7%)	1 (12.5%)	2 (25.0%)
Dermatologicals	2 (3.0%)	0	0	0	0	1 (6.7%)	0	1 (12.5%)
Genito-urinary system and sex hormones	0	0	1 (5.9%)	0	0	2 (13.3%)	1 (12.5%)	0
Musculo-skeletal system	0	1 (2.8%)	1 (5.9%)	0	0	3 (20.0%)	2 (25.0%)	1 (12.5%)
Nervous system	0	2 (5.6%)	2 (11.8%)	3 (13.6%)	0	2 (13.3%)	2 (25.0%)	1 (12.5%)
Respiratory system	5 (7.6%)	3 (8.3%)	0	1 (4.5%)	1 (7.1%)	0	1 (12.5%)	0
Sensory organs	2 (3.0%)	0	0	0	0	0	0	1 (12.5%)
Systemic hormonal preparations (excluding sex hormones and insulins)	2 (3.0%)	0	0	0	0	1 (6.7%)	0	0

*ATC* Anatomical Therapeutic Chemical, *max* Maximum, *min* Minimum, *SD* Standard deviation

#### Healthcare professional visits

**Fig. 3:**
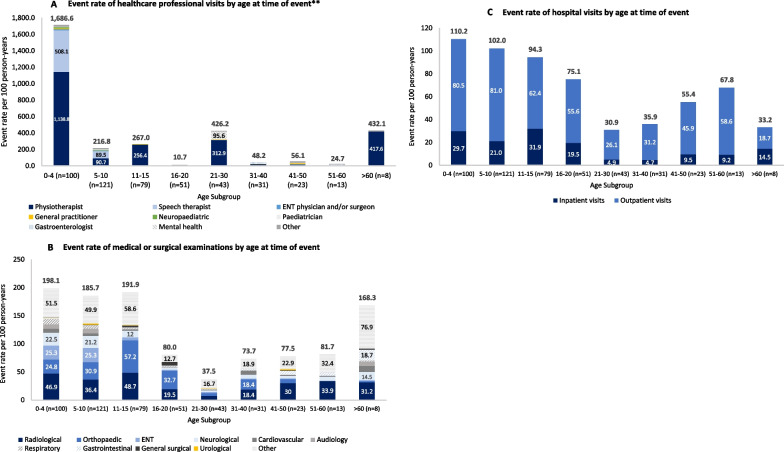
Healthcare resource use by age subgroup. Event rates per 100 person-years were calculated as the number of events divided by the total number of patient historical years multiplied by 100. **Results are displayed for healthcare professional types with an event rate of > 10 per 100 person-years in at least one age category. ‘Other’ healthcare professional visits include those to cardiologists, dentists, dermatologists, dieticians, emergency doctors, endocrinologists, geneticists, gynaecologists, neurologists, neurosurgeons, occupational therapists, ophthalmologists, orthopaedic physicians or respiratory physicians. *ENT* Ear, nose and throat

Prior to enrolment, visits to any healthcare professionals were recorded in the case report form for 60.8% of patients. A total of 18,561 visits were reported at a rate of 778.4 events per 100 person-years. The most frequent visits were with physiotherapists (569.6 visits per 100 person-years; 31.7%) and speech therapists (163.0 visits per 100 person-years; 13.4%). Overall, visits to healthcare professionals occurred substantially more frequently before the age of 5 years and visits to physiotherapists and speech therapists were most frequent in this age group (Fig. 3A). Gastroenterologists were seen most frequently between the ages of 31–40 and 51–60 years, and mental health professionals were seen most frequently by individuals aged 21–30 years (Fig. 3A). For other types of healthcare professional, there were no substantial differences between age groups.

Patients without limb lengthening procedures had a greater overall frequency and rate of visits to healthcare professionals compared to patients with limb lengthening procedures (975.0 per 100 person-years [66.4% of patients] vs 267.9 per 100 person-years [40.0% of patients]; Table 3). This difference is largely explained by the higher rate of visits to physiotherapists among patients without limb lengthening (751.9 per 100 person-years vs 96.4 per 100 person-years). Differences between other types of healthcare professional visit were negligible.

#### Medical or surgical examinations

Almost all patients (99.5%) had at least one examination to inform their medical or surgical management prior to enrolment in the study. A total of 3,312 examinations were reported, at a rate of 138.9 events per 100 person-years. The most common examinations were radiological (30.9 per 100 person-years; 69.9%), orthopaedic (25.7 per 100 person-years; 54.3%), ENT (14.8 per 100 person-years; 43.5%) and neurological (12.7 per 100 person-years; 39.2%).

Medical or surgical examinations occurred at all ages, though were reported at higher frequencies before the age of 15 years and after the age of 60 years (Fig. 3B). ENT, neurological, cardiovascular, audiology and respiratory examinations occurred more frequently before the age of 10 years, while orthopaedic examinations occurred more frequently before the age of 20 years. For those patients with data from the age of > 60 years (*N* = 8), medical or surgical examinations occurred across a broad range of specialities.

All patients (100%) with limb lengthening had undergone at least one medical or surgical examination prior to enrolment compared to 99.3% of patients without limb lengthening (Table 3). However, the rate of medical or surgical examination events was numerically lower among patients with limb lengthening (123.8 per 100 person-years vs 144.7 per 100 person-years, respectively).

#### Hospital visits

Most patients (72.0%) reported at least one inpatient hospital visit, at a rate of 22.4 per 100 person-years. Of these events, 39.6% were related to surgical procedures and 26.8% were related to medical treatment. The mean and median lengths of inpatient hospital stays were 6.7 days and 4.0 days, respectively. Hospital stays were generally longer in older age groups, with the longest mean (SD) stays seen in the group aged > 60 years (20.5 [10.9]) and shorter stays (means ranging from 5.0 to 8.2 days) for patients aged 0–30. In general, inpatient visits were reported among a greater proportion of patients, and at higher rates, before the age of 20 years than after this age (Fig. 3C).

### Patient-reported outcomes

#### Quality of life (children [aged 5–17 years])

**Fig. 4:**
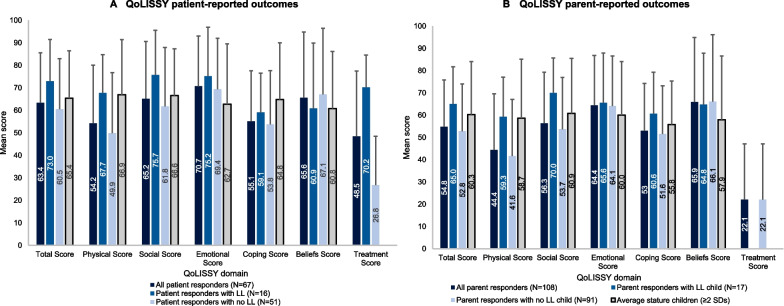
QoLISSY results (patients aged 8–17 years; parents of patients aged 5–17 years; subset of study population). QoLISSY scores for each domain range from 0 to 100 (where higher scores indicate better QoL). Empty bars correspond to missing (not available/reported) data, rather than scores of 0. Error bars are SD. Published QoLISSY scores for age-matched average stature (individuals with a height no more than 2 SDs below average height) children and parents of average stature children are also provided [24]. *LL* limb lengthening, *QoLISSY* Quality of Life in Short Stature Youth, *SD* Standard deviation

The QoLISSY questionnaire was completed by 67 children (8–17 years old; 16 with limb lengthening and 51 without) and 108 parents of children aged 5–17 years (17 patients with limb lengthening and 91 without). Parent-reported scores were lower than patient-reported scores across the majority of domains, but in both groups the lowest scores were reported in the Physical and Coping domains (Fig. 4). The patient- and parent-reported scores for all respondents were lower than average stature individuals (in this case defined as individuals < 2 SDs below average height) [24] across the majority of domains, and particularly in the Physical domain. In general, patient- and parent-reported scores were higher for individuals with limb lengthening compared to those without.

**Fig. 5:**
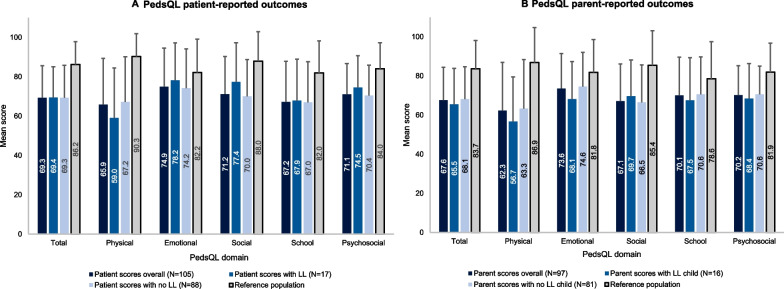
PedsQL results (patients aged 5–17 years; parents of patients aged 5–17 years; subset of study population). PedsQL scores for each domain/summary scale range from 0 (poor health-related QoL) to 100 (perfect health-related QoL). Published PedsQL scores for average stature populations are also provided. Error bars are SD. Published PedsQL scores for reference populations are also provided [25]. *LL* limb lengthening, *PedsQL* Pediatric Quality of Life Inventory, *SD* Standard deviation

The PedsQL questionnaire was completed by 105 children (5–17 years old; 17 with limb lengthening and 88 without) and 97 parents of patients aged 5–17 years (16 patients with limb lengthening and 81 without). Across both patient- and parent-completed questionnaires, the lowest mean (SD) domain scores were observed for Physical Functioning and School Functioning (Fig. 5). Across all domains, both patient- and parent-reported scores were lower than those of age-matched average stature individuals, [25] particularly in the Total and Physical Functioning domains. Patients with limb lengthening reported numerically higher scores across all domains except the Physical domain. However, contrasting trends were seen for parent-reported scores, which were higher for those without limb lengthening for all domains except the Social domain.

#### Quality of life (adults [patients aged ≥ 18 years])

**Fig. 6:**
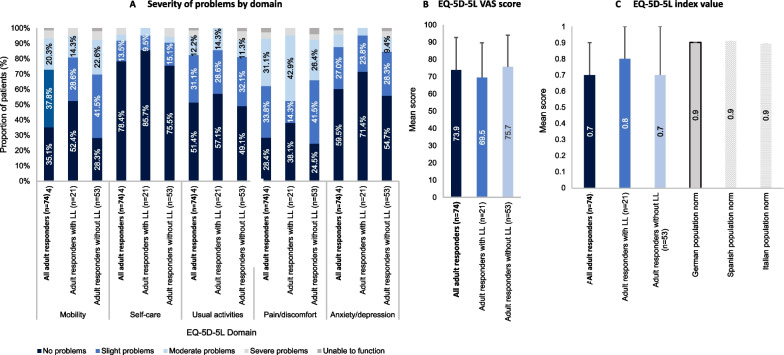
EQ-5D-5L results (patients aged ≥ 18 years; subset of study population). The EQ-5D-5L measure is comprised of two sections: the first assesses five domains impacting quality of life (mobility, self-care, usual activity, pain/discomfort and anxiety/depression). Combining the levels for each domain creates the overall index value. The second section measures self-rated (global) health status utilising a vertically oriented visual analogue scale (VAS) where 100 represents the “best imaginable health state” and 0 represents the “worst imaginable health state.” Error bars are SD. Population norms are provided for the EQ-5D-5L index values for Germany (0.902), Spain (0.915) and Italy (0.899) [26]. *SD* Standard deviation, *VAS* Visual analogue scale

A total of 74 adults completed the EQ-5D-5L questionnaire (21 with limb lengthening and 53 without). Figure 6 presents the reported severity of problems by domain (from no problems to unable to function; Fig. 6A), the EQ-5D-5L visual analogue scale (VAS; Fig. 6B) scores and the EQ-5D index scores (Fig. 6C). Index scores are also presented for the general populations of Germany, Italy and Spain. The domain with the greatest proportion of patients reporting moderate to extreme problems was pain/discomfort (37.9%), followed by mobility (17.7%). The mean (SD) VAS score was 73.9 (18.9), and the mean (SD) index score was 0.7 (0.2). Adult patients that had undergone limb lengthening reported higher mean ED-5D index values than those that had not. All subgroups of the study population, including those who had undergone limb lengthening, reported lower mean EQ-5D index values than those of the general populations of Germany, Italy or Spain (**C**) [26].

**Fig. 7:**
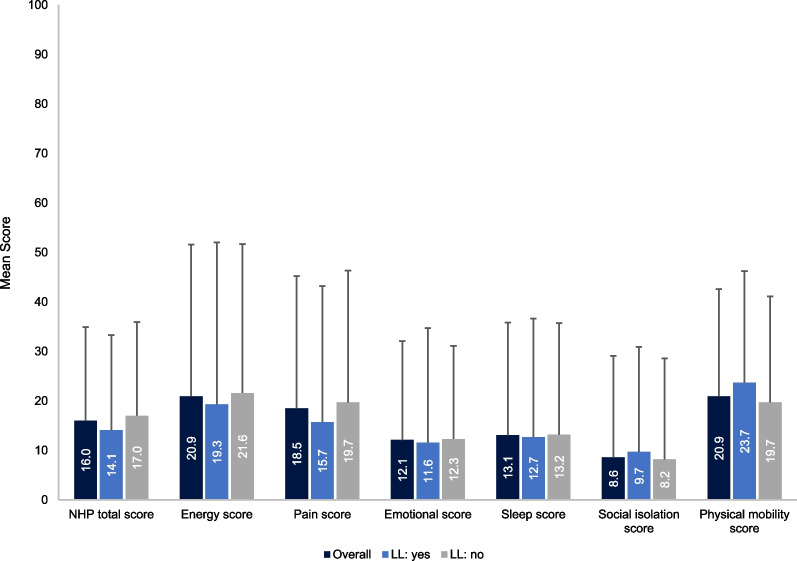
NHP results (patients aged ≥ 18 years; subset of study population). *NHP scores for each domain range from 0 to 100 (with 0 indicating no distress [good subjective health] and 100 indicating severe distress [poor subjective health]). Error bars are SD. *LL* Limb lengthening*, NHP* Nottingham Health Profile, *SD* Standard deviation

The NHP questionnaire was completed by 74 adults (21 with limb lengthening and 53 without). The most negatively impacted domains were for Physical Mobility, Energy and Pain (Fig. 7). For Energy, Pain and Total scores, patients with limb lengthening reported lower scores (better QoL) than those without limb lengthening. For the Physical Mobility domain, patients without limb lengthening reported lower scores.

#### Pain (patients aged ≥ 8 years)

The APPT questionnaire was used to assess pain among patients aged 8–17 years. In total, 50 patients completed the APPT questionnaire (13 with limb lengthening and 37 without). Of these, 41 patients responded to questions about the number of pain sites; 58.6% reported at least one pain site and 32.9% reported ≥ 3 pain sites. 43 patients responded to questions about pain intensity; pain intensity was rated “little pain” by 34.9% and “medium pain” by 27.9% of patients. The most frequent pain site locations were the knees and lower spine. Patients without limb lengthening had a greater mean number of pain sites and a slightly higher total pain score than those with limb lengthening.

The BPI-SF was used to assess pain among patients aged ≥ 18 years old. The questionnaire was completed by 72 patients (20 with limb lengthening and 52 without), 70.3% of whom reported at least one pain site and 41.9% reported ≥ 3 pain sites. Overall, 62 patients responded to the question about pain severity; the mean (SD) pain severity score (where zero indicates no pain and ten indicates the worst pain imaginable) was 2.9 (2.2). In addition, 63 patients responded to the question about how pain interferes with everyday life; the mean (SD) interference score (where zero indicates no interference and ten indicates complete interference) was 2.5 (2.5). The most frequent pain site locations were the knees and lower spine. Adult patients who had received limb lengthening reported higher severity and intensity scores (indicating worse pain) than those who had not undergone these procedures.

#### Functional independence (children [patients aged 5–17 years])

Functional independence among patients aged 5–17 years old was assessed using the WeeFIM questionnaire, which was completed by a total of 104 patients (16 with limb lengthening and 88 without). The mean (SD) Total score, which has a score range of 18 (total assistance) to 126 (complete independence), was 112.7 (13.3). The mean (SD) score for the Mobility domain, which has a score range of 5 (total assistance) to 40 (complete independence) was 31.4 (4.6). The mean (SD) score for the Cognition domain (which had the same score range as the Mobility domain) was 33.6 (2.9). The mean (SD) Self-Care score, which has a score range of 5 (total assistance) to 56 (complete independence) was 47.7 (8.4).

Patients with limb lengthening reported numerically higher mean (SD) Total, Mobility and Self-Care scores than those without limb lengthening (Total: 118.8 [9.0] vs 111.5 [13.7]; Mobility: 33.6 [2.9] vs 31.0 [4.8]; Self-Care: 51.8 [6.1] vs 46.9 [8.5], respectively). Cognition scores were similar between the two groups (33.4 [2.8] vs 33.6 [2.9], respectively).

#### Work productivity and activity impairment (adults [patients aged ≥ 18 years])

Overall, 73 patients aged ≥ 18 years completed the WPAI questionnaire (20 with limb lengthening and 53 without). Of these, 30 (41.1%) were unemployed. Among the 43 subjects who were in paid employment, the mean (SD) number of hours missed from work in the past week due to problems associated with achondroplasia was 1.8 (6.9) and the mean (SD) number of hours missed due to any other reason (e.g., vacation, holidays, time off to participate in this study) was 2.5 (6.1). Overall, the mean number of hours actually worked during the past week was 36.6 (13.9) and the mean percentage of work time missed due to achondroplasia was 5.1% (19.8%). Patients without limb lengthening missed a greater mean percentage of work time due to achondroplasia compared to those with limb lengthening (6.4% [22.9%] vs 1.6% [4.9%]).

The mean percentage of impairment while working due to achondroplasia (impact on productivity over the past week) was 15.4% (23.3%) and the mean percentage of activity impairment due to achondroplasia (impact on the ability to do regular daily activities, other than work at a job, over the past week) was 26.6% (25.4%). Patients who had not undergone limb lengthening reported greater impairment compared to those who had (18.6% [25.3%] vs 5.6% [11.3%] for work-related activities, and 27.3% [25.4%] vs 24.7% [25.9%] for regular daily activities other than work).

#### Exploratory analysis of the relationship between height and patient-reported outcomes (QoL, pain and functional independence)

The baseline characteristics of the sub-set of patients (N = 125) included in the exploratory analysis is presented in Additional File 7. There was a mean age of 20.88 years at the date of the most recent height measurement (mean age of 22.15 at the date of the patient-reported outcomes questionnaire) and a slightly greater proportion of female than male patients (55.2% vs 44.8%). Eligible patients who had undergone limb lengthening were included in the sub-set.

*Significant positive correlations were observed between height z-score and QoL in patients 5–17 years old, as measured by patient-reported QoLISSY and PedsQL total scores (QoLISSY: 0.394 [p* ≤ *0.01]; PedsQL: 0.266 [p* ≤ *0.01]). Similarly, height was significantly positively correlated with the QoLISSY total score in this group (Table **5*

**Table 5:** Correlation analysis between height/z-score and patient-reported outcomes (subset of study population)

Measure	Height^a^	Height z-score
*QoLISSY*^*b*^* (administered to patients 5–17 years old; n = 67)*		
Physical score	0.387*	0.466*
Social score	0.366*	0.345*
Emotional score	0.203	0.229
Coping score	0.052	− 0.230
Beliefs score	0.159	0.324*
Future score	0.117	0.268*
Effects score	0.232	0.059
Total score	0.361*	0.394*
*PedsQL*^*b*^* (administered to patients 5–17 years old; n = 63)*		
Physical score	0.041	0.273*
Emotional score	0.054	0.100
Social score	0.200	0.177
School score	− 0.054	0.255*
Psychosocial score	0.076	0.218
Total score	0.068	0.266*
*EQ-5D-5L*^*c*^* (administered to patients ≥ 18 years old; n = 55*)		
Mobility score	− 0.039	− 0.065
Self-care score	− 0.118	− 0.167
Usual activities score	− 0.184	− 0.157
Pain/discomfort score	0.134	0.102
Anxiety/depression score	− 0.206	− 0.263*
Utility	0.083	0.143
VAS score	− 0.180	− 0.158
*APPT*^*d*^* (administered to patients 8–17 years old; n = 18*)		
Sensory score	0.292	− 0.122
Affective score	0.547*	0.156
Evaluative score	0.147	0.212
Temporal score	0.538*	0.233
Total pain score	0.641*	0.191
*BPI-SF*^*d*^* (administered to patients ≥ 18 years old; n = 45*)		
Severity score	0.078	0.079
Interference score	0.009	− 0.050
*WeeFIM*^*d*^* (administered to patients 5–17 years old; n = 58*)		
Self-care score	0.423*	0.339*
Mobility score	0.399*	0.404*
Cognition score	0.076	0.036
Total score	0.407*	0.351*

^*^Indicates significant results (*p* < 0.05 or *p* < 0.01)

^a^Positive cut-off value indicates a positive correlation. Negative cut-off value indicates a negative correlation. Coefficients included Pearson’s and Spearman’s. Very high correlation: 0.9─1.0; high correlation: 0.7─ < 0.9; moderate correlation: 0.5─ < 0.7; low correlation: 0.3─ < 0.5; negligible correlation: 0.0─ < 0.3. As exploratory analyses, it cannot be concluded that any *p*-values or statistical significance imply causality

^b^Self-assessment (if available), otherwise caregiver proxy assessment; 3/67 and 2/61 of the questionnaries for the QoLISSY and PedsQL were completed by proxies, respectively

^c^A lower score on the EQ-5D-5L indicates better QoL; therefore, a negative correlation with the EQ-5D score indicates a positive association with QoL

^d^Proxy-assessment

*APPT* Adolescent Pediatric Pain Tool, *BPI-SF* Brief Pain Inventory-Short Form, *PedsQL* Pediatric quality of life inventory, *QoL* Quality of life, *QoLISSY* Quality of Life in Short Stature Youth, *WeeFIM* Paediatric Functional Independence Measure, *VAS* Visual analogue scale

. In patients ≥ 18 years old, there were no significant and meaningful correlations observed between height z-score/height and results of the EQ-5D-5L questionnaire. Overall, the results of the OLS regression analyses (Additional File 7) identified height z-score to be a significant driver of the QoLISSY total score (*p* < 0.01), as well as the PedsQL total score (*p* < 0.05).

No notable correlations were identified between height z-score and pain among patients aged 8–17 years old, as assessed by the APPT questionnaire. However, height was shown to be significantly positively correlated with the APPT total pain score (0.641 [*p* ≤ 0.01]). Neither height z-score nor height were significantly correlated with pain severity or interference scores from the BPI-SF in patients aged ≥ 18 years (Table 5).

A significant positive correlation between height z-score and the total score of the WeeFIM questionnaire was identified among patients aged 5–17 (0.351 [*p* ≤ 0.01]). The results of the correlation analysis between height and total WeeFIM score supports this finding (0.407 [*p* ≤ 0.01]; Table 5).

## Discussion

LIAISE was a multinational, retrospective, observational natural history study designed to quantify the medical, functional, QoL, socio-economic and resource-related impacts of achondroplasia across the lifespan of patients. Patients of all ages were enrolled into the study and the retrospective design allowed collection of data from medical records for a considerable period of time prior to enrolment.

Overall, a high number and broad range of complications were reported across age groups, supporting previous studies which highlighted the multisystemic complication burden associated with achondroplasia [3, 5, 27]. Complications were particularly frequent at the youngest and oldest ages, with event rates following a U-shaped distribution across the study population. Under the age of 10 years, the increased event rate was predominantly explained by a greater rate of foramen magnum stenosis, likely among individuals < 2 years old as suggested by existing literature [3]. Meanwhile, increased rates of ENT issues (e.g. sleep apnoea, ear disorders, pharyngitis), other orthopaedic problems (e.g. musculoskeletal disorders, osteoarthritis, joint injury), spinal cord compression/stenosis, pain, and gastroesophageal issues (e.g. inflammatory bowel disease, gastroesophageal reflux disease, dyspepsia and tooth abscess) were observed at ages above 40 years. While pain also tends to increase with age among the general population, [28–32] it may be exacerbated among individuals with achondroplasia due to long-term spinal stenosis, joint problems, headaches or other ENT-related problems.

The varied complication burden across patient lifespan appeared to lead to differing healthcare needs and priorities, which was reflected in the healthcare resource use data of the study population. The high burden of complications in early life appeared to be associated with a high rate of surgeries. There were particularly high rates of limb lengthening among adolescents from countries where these procedures are more common [33–35]. There was also a high rate of brainstem decompressions and surgeries to improve breathing and hearing among 0–10 year-olds. This is in line with clinical guidance that foramen magnum stenosis and ENT complications often require early surgical intervention [36–38]. Meanwhile, complications in mid-to-late adulthood appeared to manifest as increased medication use, with notable increases in the prescription of medications relating to musculoskeletal, neurological, gastrointestinal and cardiovascular systems. Healthcare resource use among the oldest age groups was lower than might be expected, given the high rates of complications among these individuals. This may be explained by a lack of structured and accessible care for older individuals with achondroplasia or a reduced capability for older patients to regularly attend appointments related to complications or mobility problems. It may also be a result of less complete medical records for the oldest age groups; given that many of the older individuals within the study were recruited via referrals or associations with patient organizations, medical records were provided by the patients themselves rather than the study investigators. Furthermore, selection bias within the representative but small LIAISE population is a plausible factor, whereby those with more health issues may have been more motivated to participate in the study.

QoL and other patient-reported outcomes data were collected from a large number of patients via self- and proxy-reported questionnaires. Many of the questionnaires indicated that individuals with achondroplasia experience challenges with mobility and pain. In addition, reduced scores in the PedsQL School domain compared to average stature individuals, together with the results of the WPAI questionnaire, suggest that the individuals in this study experience challenges at school and work, highlighting the need for improvements in school and work settings to appropriately support the needs of these individuals. Where comparisons with average stature individuals were possible (QoLISSY, PedsQL and EQ-5D-5L questionnaires), patients with achondroplasia reported lower scores across the majority of domains. However, the opposite was true for the patient-reported scores in the QoLISSY Emotional and Beliefs domains. This may be explained by recent findings that individuals with achondroplasia can also have positive experiences as a result of their condition, including the receipt of positive attention and having a supportive achondroplasia community [9]. For both the QoLISSY and PedsQL, self-reported scores were typically higher than parent-reported scores, indicating a difference in the perception of QoL between parents and children, whereby parents consider the QoL of their child to be more detrimentally impacted than the patients themselves do. This trend is supported by similar findings from a previous study [39]. While the cause of such differences remains to be investigated, these results indicate the need to apply caution when using parent-reported scores as proxies for patient QoL.

The exploratory analysis found several weak but positive correlations between height and patient-reported outcomes. In general, the disease-specific tools (QoLISSY/WeeFIM) appeared to be more often correlated with height and height z-score than the generic tools (EQ-5D-5L, PedsQL), suggesting that the use of disease-specific tools may be more appropriate when assessing QoL in individuals with achondroplasia. The moderate correlations identified between height outcomes and QoL in younger patients, as measured by the QoLISSY and PedsQL questionnaires, are in line with previously reported associations between these variables and highlight the potential for height outcomes to be used as a proxies for QoL [10, 11]. Lastly, the observed correlation between height outcomes and functionality, particularly in the Mobility domain, suggests that there may be a relationship between improved height and improved mobility and physical functioning. These may be valuable insights in the context of available and emerging precision therapies for achondroplasia, though the causality of such relationships requires further investigation.

A total of 40 patients had undergone limb lengthening prior to study enrolment. The majority were recruited from study sites in Italy and Spain, as was expected due to the increased practice of limb lengthening in these countries. Most patients with limb lengthening were female; however, this does not appear reflective of other published limb lengthening data and may be a result of recruitment biases [40]. The differences between patients with and without limb lengthening with regards to the observed occurrence of complications, healthcare resource needs and patient-reported outcomes encourages further research to be conducted in this area among larger study populations.

### Limitations

Despite a large overall sample, the LIAISE study population was associated with limitations. For example, some countries were represented only by a small number of patients, there was a general overrepresentation of female patients and it was difficult to recruit patients over the age of 30 years. The duration of available medical data also varied between patients. Further, while the number of patients with a history of limb lengthening was capped at 20% to minimise selection bias, the subgroups of patients who did or did not have limb lengthening differed in size and demographics, reducing the capacity to conduct robust comparisons between these two groups. Patients with limb lengthening were recruited predominantly from just two countries. Across all outcomes, data were drawn and combined from patients across several countries, which have varied health and social care practices, patient associations and disease management approaches.

Though the use of a pre-defined electronic case report form allowed data collection to focus on outcomes considered to be the most relevant to the achondroplasia population, this method also limited the capacity for investigators to easily specify other (potentially unexpected) impacts of the condition. In addition, since the case report form was written in English, investigators without proficiency in English may have interpreted the terminology differently. Any missing events due to incomplete medical records, for example if events occurred at a different healthcare centre, may have led the event rates of some outcomes to be under-reported. Patient-reported outcomes were based on self-reported questionnaire data which is associated with potential bias and a lack of generalisabilty, particularly given that the proportion of patients (or parents) who provided complete responses varied between questionnaires.The causality of correlations between height z-score/height and patient-reported outcomes remains unclear, and the robustness of such correlations are limited by the smaller sample size, the different timepoints at which the height and patient-reported outcome measurements were made and the inclusion of patients who had received limb lengthening surgery in these exploratory analyses. Additional exploratory analyses between patient-reported outcomes and other clinical or demographic features were not presented.

## Conclusion

The findings of this retrospective, observational, natural history study suggest that individuals with achondroplasia experience a range of serious complications throughout their lives resulting in a high level of healthcare resource needs and reduced QoL compared to unaffected reference populations. Such results provide important insights into the medical experiences of individuals with achondroplasia throughout their lives, as well as the current clinical practices across Europe. This study indicates several areas for potential future research. It also demonstrates the utility of retrospective study designs in understanding the natural history of rare conditions. Importantly, LIAISE revealed varied approaches to achondroplasia management, and highlights the value of an international consensus on management practices [41] to ensure high quality care for all patients across geographical regions.

## Supplementary Information

**Additional file 1:** List of data categories collected from patient medical records.

**Additional file 2:** List of pre-defined complications of interest by relevant age population.

**Additional file 3:** Table of descriptions, including ages assessed, by each patient-reported outcomes questionnaire.

**Additional file 4 :** Imputation methods for missing data.

**Additional file 5:** Methods for exploratory analyses between height/z-score and patient-reported outcomes.

**Additional file 6:** Growth charts (height and head circumference) of study population against published achondroplasia growth data, by time of measurement, age and sex.

**Additional file 7:** Baseline characteristics of the study population included in the exploratory analyses and full OLS regression models between independent variables (height z-score, absolute height [cm], age, gender and complications) and patient-reported outcomes (total scores).

## Data Availability

The data for the research presented in this publication may be available from the corresponding author upon reasonable request.
