# Supplementary material for: Lifetime impact of achondroplasia study in Europe (LIAISE): findings from a multinational observational study
Source: Orphanet J Rare Dis. 2023 Mar 15;18:56. doi: 10.1186/s13023-023-02652-2 (PMC10015810; doi:10.1186/s13023-023-02652-2)
Supplement: Supplementary file 1 — Additional file 1: List of data categories collected from patient medical records. [file 13023_2023_2652_MOESM1_ESM.docx]

**Additional File 1: Data categories collected from patient medical records**

- Date of data collection
- General demographics (e.g., date of birth, age, gender)
- Date of diagnosis of achondroplasia
- Diagnosis of achondroplasia (genetic confirmation of achondroplasia; clinically confirmed, by specialist, through clinical examination or radiological assessment)
- Clinical characteristics of achondroplasia at diagnosis (i.e., disproportionate short stature, rhizomelic shortening of the arms and legs, short fingers, limitation of elbow extension, trident configuration of the hands, bow legs, thoracolumbar kyphosis, exaggerated lumbar lordosis, large head with frontal bossing, mid-facial hypoplasia, depressed nasal bridge)
- Radiological characteristics of achondroplasia at diagnosis (i.e., long bones, spine, pelvis, femurs)
- Molecular genetic testing (date, confirmation of achondroplasia, type of mutation on the FGFR3 gene)
- Anthropometric measurements: growth characteristics at different ages (i.e., height, weight, head circumference, proportionality of body parts)
- Mobility tests (dates and results)
- Medical history (investigators selected categories from a drop-down list; categories included macrocephaly/hydrocephalus, obstructive sleep apnea, respiratory disorders, cardiovascular disorders, middle ear dysfunction, speech delay, adenotonsillar hypertrophy, dental overcrowding, bowing of the lower legs, kyphosis at the thoracolumbar junction, foramen magnum compression, symptomatic spinal stenosis/cord compression, arthritis/rheumatism, chronic neck or back pain, psychological disorders, musculoskeletal disorders, neurological disorders, obesity, ENT issues, gastroenterology disorders, endocrinology disorders, other)
- Surgical history (investigators inputted dates and selected categories from a pre-defined list; categories included limb lengthening, spinal correction, grommet insertion, shunt insertion, shunt revision, brainstem decompression, tonsillectomy/adenoidectomy, orthodontic procedures, osteotomy, nerve decompression, myringotomory, tympanostomy with tube placement)
- Treatment and procedural history (e.g., dates and type of treatments: medication prescriptions, continuous positive airway pressure or other ventilation support, speech therapy, family support)
- Dates and results of previous examinations (investigators selected categories from a drop-down list; categories included radiological, cardiovascular, neurological, audiology, other)
- Dates, reasons and results of tests from previous visits to other healthcare professionals (investigators selected categories from a pre-defined list; categories included geneticist, paediatrician, respiratory physician, neurologist, neurosurgeon, physiotherapist, occupational therapist, speech therapist, dietician, orthoptist)
- Healthcare resource use for a minimum of the five years prior to the date of enrolment (number of health-professional visits, hospitalisations, emergency room visits)
- History of pregnancies and their outcomes (female patients of childbearing potential)
