# Supplementary material for: Lifetime impact of achondroplasia study in Europe (LIAISE): findings from a multinational observational study
Source: Orphanet J Rare Dis. 2023 Mar 15;18:56. doi: 10.1186/s13023-023-02652-2 (PMC10015810; doi:10.1186/s13023-023-02652-2)
Supplement: Supplementary file 2 — Additional file 2: List of pre-defined complications of interest by relevant age population. [file 13023_2023_2652_MOESM2_ESM.docx]

**Additional File 2: Pre-defined complications of interest [relevant age population]**

- Otitis media [for patients aged ≤10 years at index]
- Other ENT issues (excluding otitis media) [all ages]
- Genu varum/vagum [for patients aged ≤20 years at index]
- Spinal deformities (e.g., kyphosis/lordosis) [all ages]
- Foramen magnum syndrome/stenosis [for patients aged ≤5 years at index]
- Spinal cord compression/stenosis [all ages]
- Other orthopaedic problems [all ages]
- Infections/infestations [all ages]
- Gastroesophageal issues [all ages]
- Pain (any type) [all ages]
