# Supplementary material for: Lifetime impact of achondroplasia study in Europe (LIAISE): findings from a multinational observational study
Source: Orphanet J Rare Dis. 2023 Mar 15;18:56. doi: 10.1186/s13023-023-02652-2 (PMC10015810; doi:10.1186/s13023-023-02652-2)
Supplement: Supplementary file 3 — Additional file 3: Table of descriptions, including ages assessed, by each patient-reported outcomes questionnaire. [file 13023_2023_2652_MOESM3_ESM.docx]

Additional File 3: Description of patient-reported outcome questionnaires

| **Questionnaire** | **Ages Assessed in LIAISE (years)** | **Description** |
| --- | --- | --- |
| **Quality of Life** | | |
| PedsQL(14) | 5–17 | 23 items assigned to three QoL dimensions: physical, social and emotional aspects of functioning, as well as school functioning |
| QoLISSY(13) | 8–17 | 22 items assigned to three QoL dimensions: physical, social and emotional aspects of functioning.  28 additional items reflecting predictors of QoL: coping, general beliefs about height and treatment-specific aspects |
| EQ-5D-5L(18) | ≥18 | Five dimensions: mobility, self-care, usual activities, pain/discomfort and anxiety/depression |
| NHP(20) | ≥18 | Two-part questionnaire which measures QoL. The first part comprises of: energy, sleep, emotions, pain, mobility and social isolation. The second assesses areas of life commonly affected by health: paid employment, household chores, social life and personal relationships, hobbies and interests, and holidays |
| **Pain** | | |
| APPT(16) | 8–17 | Instrument for self-reporting of pain by 8–17-year olds, composed of three parts: body diagram to mark pain location; line scale from ‘no pain’ to ‘worst possible pain’ to measure pain intensity; 56 word list to assess sensory, affective, evaluative and temporal dimensions of pain |
| BPI-SF(19) | ≥18 | Assesses clinical pain in terms of severity and interference with general activity, mood, walking ability, normal work, social relations, sleep, and enjoyment of life |
| **Functional Independence** | | |
| WeeFIM(15) | 5–17 | Measures the need for assistance and the severity of disability in children by assessing physical function and activity impact. 18 items covering six domains: self-care, sphincter control, mobility, locomotion, communication, and social cognition |
| **Work Productivity and Impairment** | | |
| WPAI:SHP(17) | ≥18 | Assesses impairments in employment and social activities for specific health problems. It measures percent of work missed, percent impairment whilst working, percent activity impairment and percent of overall work impairment due to a problem. WPAI outcomes are expressed as impairment percentages, with higher numbers indicating greater impairment and less productivity. |

**Abbreviations:** APPT: Adolescent Pediatric Pain Tool; BPI-SF: Brief Pain Inventory-Short Form; NHP: Nottingham Health Profile; PedsQL: pediatric quality of life inventory; QoL: quality of life; QoLISSY: Quality of Life in Short Stature Youth; WeeFIM: paediatric Functional Independence Measure; WPAI:SHP: Work Productivity and Activity Impairment Questionnaire for a Specific Health Problem.

**Sources:** Bullinger et al. 2013;(13) McCabe and Granger, 1990;(15) Hunt et al. 1995;(20) Cleeland and Ryan, 1994;(19) Reilly et al. 1993;(17) The EuroQol Group 2009;(18) Varni 1999;(14) Wilkie et al. 1990.(42)
