# Supplementary material for: Lifetime impact of achondroplasia study in Europe (LIAISE): findings from a multinational observational study
Source: Orphanet J Rare Dis. 2023 Mar 15;18:56. doi: 10.1186/s13023-023-02652-2 (PMC10015810; doi:10.1186/s13023-023-02652-2)
Supplement: Supplementary file 4 — Additional file 4 : Imputation methods for missing data. [file 13023_2023_2652_MOESM4_ESM.docx]

Additional File 4: Planned imputation

- Dates prior to enrolment with missing month and/or day information were imputed; missing year data were not imputed. Dates with a missing day were imputed as to the middle of the occurring month. Dates with missing month and day were imputed as 1^st^ July of the occurring year. Partial birthdates were imputed as 1^st^ July if only the year was present and the first day of the month if only month and year were present.
- The imputation planned for missing values for patient-reported outcome data was as follows:
  - For Brief Pain Inventory Short Form (BPI-SF) results, the mean severity score was set to missing if any pain severity question was missing. If 4 or more of the 7 pain interference questions were missing, the mean score was set to missing.
  - For Quality of Life of Short-Stature Youth (QoLISSY) questionnaire results, the sub-scale score and total score were set to missing if less than 80% of all items in a sub-scale were reported.
  - For PedsQL results, each score was set to missing if more than 50% of the items in the scale were missing.
