# Supplementary material for: Lifetime impact of achondroplasia study in Europe (LIAISE): findings from a multinational observational study
Source: Orphanet J Rare Dis. 2023 Mar 15;18:56. doi: 10.1186/s13023-023-02652-2 (PMC10015810; doi:10.1186/s13023-023-02652-2)
Supplement: Supplementary file 5 — Additional file 5: Methods for exploratory analyses between height/z-score and patient-reported outcomes. [file 13023_2023_2652_MOESM5_ESM.docx]

Additional File 5: Methods of exploratory statistical analyses

Associations were illustrated graphically using scatter plots, including trend lines estimated using ordinary least square (OLS) regression, for continuous variable pairs, and box plots for dichotomous and continuous variables, the latter showing the sample median, the first and third quartile, the minimum (Q1-1.5×IQR), and the maximum (Q1+1.5×IQR).

The following variables and measures were modelled (as dependent variables) as part of the regression analysis:

- QoLISSY (total and domain scores);
- EQ-5D (total scores);
- WeeFIM (total and domain scores);
- PedsQL (total and domain scores); and
- Comorbidities with height data within ±1 year (results not presented within this manuscript)

The following explanatory, independent variables were included in each model of quality of life measures:

- Age;
- Sex; and
- Height or height z-score (based on qualitative assessment of the strength and significance of the estimated correlation coefficients between the two variables and each modelled outcome, respectively)
