# Supplementary material for: Lifetime impact of achondroplasia study in Europe (LIAISE): findings from a multinational observational study
Source: Orphanet J Rare Dis. 2023 Mar 15;18:56. doi: 10.1186/s13023-023-02652-2 (PMC10015810; doi:10.1186/s13023-023-02652-2)

Additional File 6: Height and head circumference for age (first, mid- and last measurement) curves of the LIASE study population plotted against published achondroplasia growth data (Neumeyer, Merker and Hagenäs, 2021)(21)

1. First height measurement; individuals aged >20 years are shown as 20 years.


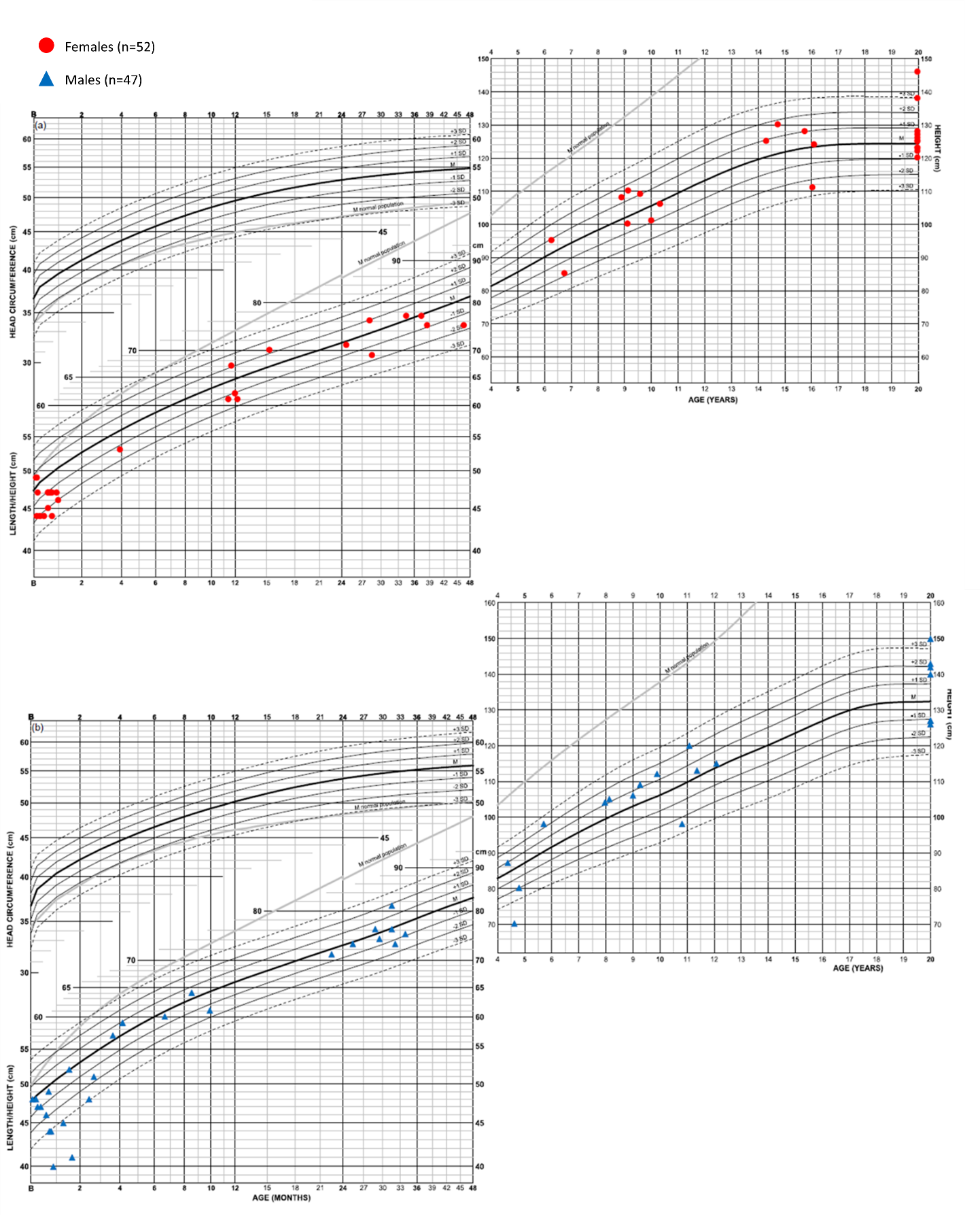


1. Mid-height measurement; individuals aged >20 years are shown as 20 years.


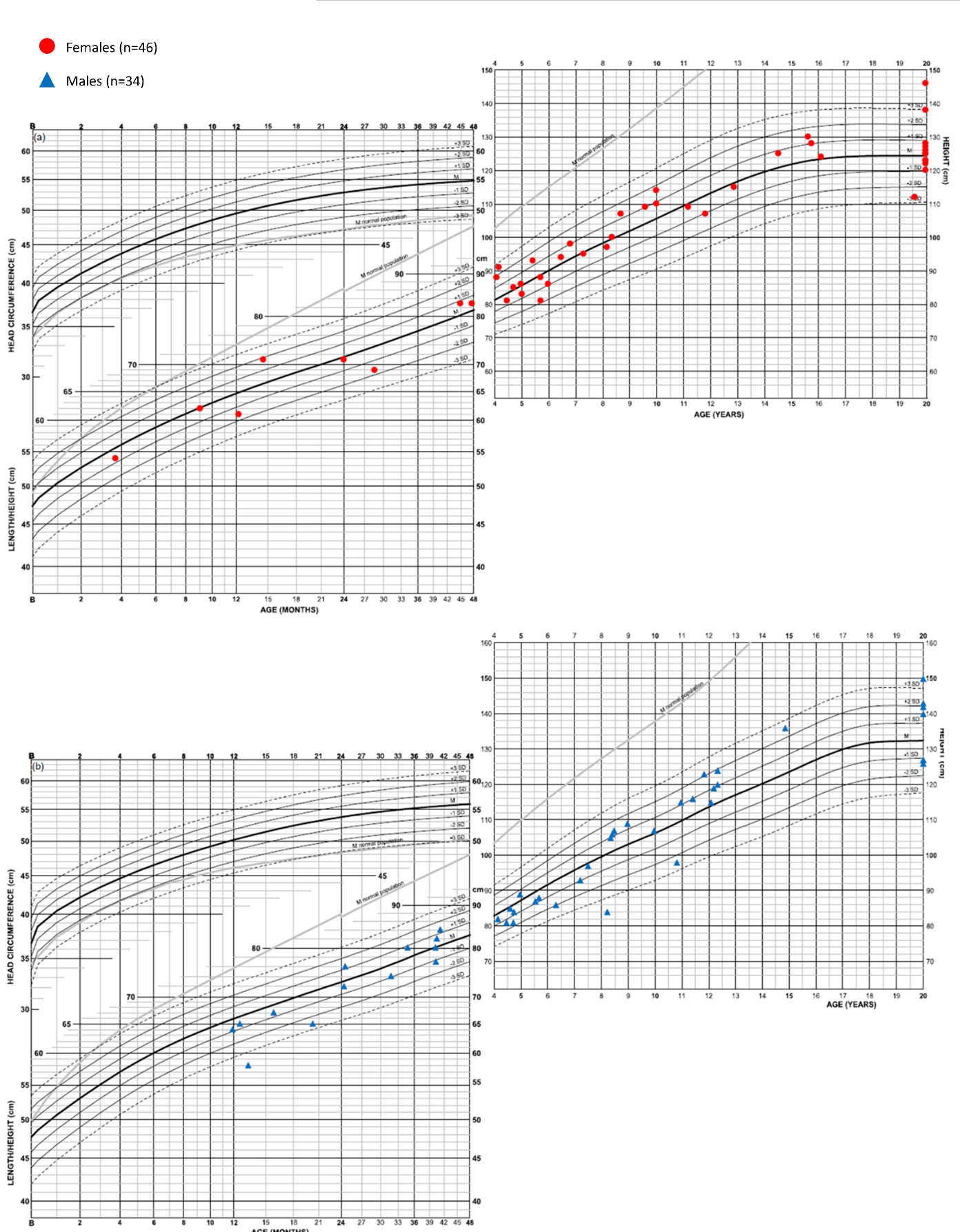


1. Last height measurement; individuals aged >20 years are shown as 20 years.


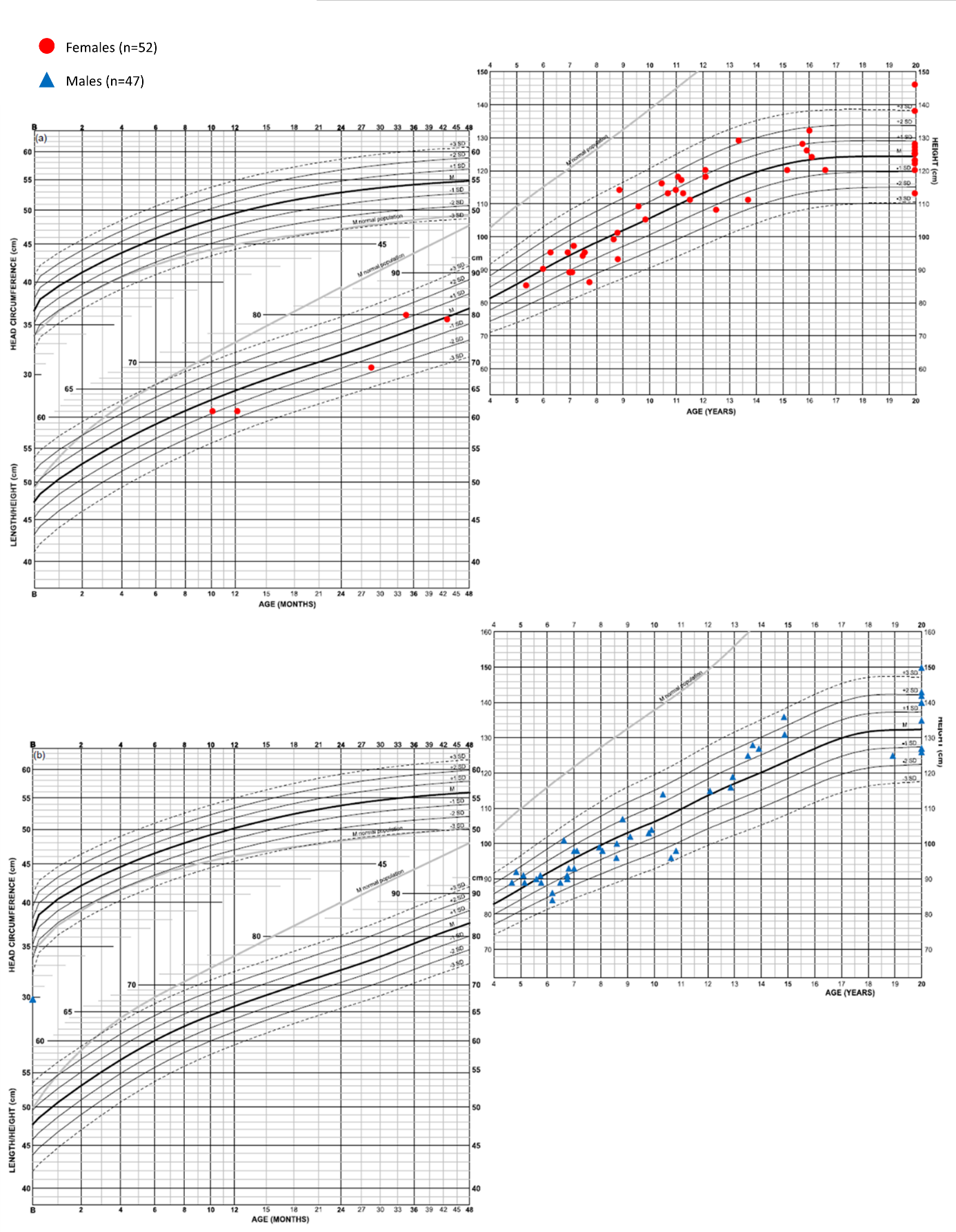

Supplement: Supplementary file 6 — Additional file 6: Growth charts (height and head circumference) of study population against published achondroplasia growth data, by time of measurement, age and sex. [file 13023_2023_2652_MOESM6_ESM.docx]
