# Supplementary material for: Lifetime impact of achondroplasia study in Europe (LIAISE): findings from a multinational observational study
Source: Orphanet J Rare Dis. 2023 Mar 15;18:56. doi: 10.1186/s13023-023-02652-2 (PMC10015810; doi:10.1186/s13023-023-02652-2)
Supplement: Supplementary file 7 — Additional file 7: Baseline characteristics of the study population included in the exploratory analyses and full OLS regression models between independent variables (height z-score, absolute height [cm], age, gender and complications) and patient-reported outcomes (total scores). [file 13023_2023_2652_MOESM7_ESM.docx]

Additional File 7: Additional results of the exploratory analysis between height and patient-reported outcomes

Baseline characteristics of the study population included in the exploratory analyses

|  | **Total sample** | **Patients <18 years old** | **Patients ≥18 years old** | **Total sample (excluding limb-lengthened)** |
| --- | --- | --- | --- | --- |
| Number of patients, n (%) | 125 (100.0) | 69 (55.2) | 56 (44.8) | 96 (76.8) |
| **Country of residence, n (%)** | |  |  |  |
| Austria | 2 (1.6) | 2 (3.0) | 0 (0.0) | 2 (2.1) |
| Denmark | 8 (6.4) | 5 (7.5) | 3 (5.1) | 7 (7.3) |
| Germany | 35 (28.0) | 18 (26.1) | 17 (30.4) | 33 (34.4) |
| Sweden | 4 (3.2) | 0 (0.0) | 4 (7.1) | 4 (4.2) |
| Italy | 48 (38.4) | 33 (47.8) | 15 (26.8) | 33 (34.4) |
| Spain | 28 (22.4) | 11 (15.9) | 17 (30.4) | 17 (17.7) |
| **Age, mean (SD) [range] years** | |  |  |  |
| At date of data collection | 22.1 (17.37) [5─68] | 9.6 (3.39) [5─17] | 37.6 (15.02) [18─68] | 20.9 (18.47)  [5-68] |
| At most recent height measurement | 20.9 (16.35) [4–68] | 9.4 (3.69) [4─17] | 35.1 (14.63) [17─68] | 19.6 (17.42) [4-68] |
| **Other characteristics** | |  |  |  |
| Sex, female (%) | 69 (55.2) | 34 (49.3) | 35 (62.5) | 44 (45.8) |
| Height, mean (SD) [range] cm | 117.9 (19.87) [84─161] | 105.3 (15.99) [84─144] | 133.5 (11.23) [113─161] | 111.9 (17.59) [84-150] |
| Height z-score, mean (SD) [range] | -5.2 (1.29) [-8 to -1] | -5.2 (0.93) [-8 to -3] | -5.1 (1.64) [-8 to -1] | -5.6 (0.94) [-8 to -3] |
| **Height z-score, n (%)** | | | |  |
| <-6 | 25 (20.0) | 8 (11.6) | 17 (30.4) | 25 (26.0) |
| -6 to -5 | 51 (40.8) | 31 (44.9) | 20 (35.7) | 44 (45.8) |
| -5 to -4 | 28 (22.4) | 25 (36.2) | 3 (5.4) | 23 (24.0) |
| -4 to -3 | 9 (7.2) | 3 (4.3) | 6 (10.7) | 3 (3.1) |
| ≥-3 | 12 (10) | 2 (2.9) | 10 (17.9) | 1 (1.0) |

**Abbreviations:** SD: standard deviation.

OLS regression analysis results: QoL/functionality with height (SDS and cm), age, gender and complications

|  | **QoLISSY**  **(total score)** | **EQ-5D-5L**  **(utility score)** | **WeeFIM**  **(total score)** | **PedsQL**  **(total score)** |
| --- | --- | --- | --- | --- |
| R^2^ | 0.36 | 0.34 | 0.49 | 0.20 |
| n | 63 | 48 | 56 | 59 |
| **Height z-score (SDS) coefficient** | 10.79*** | 0.01 | - | 5.54** |
| **Height (cm) coefficient** | - | - | 0.36* | - |
| **Age (years)** | 2.56*** | 0.00 | -0.07 | 0.41 |
| **Gender (female)** | -1.70 | -0.02 | -5.14** | -3.46 |
| **Complications** | - | - | - | - |
| Otitis media | - | - | - | - |
| ENT issues (excluding otitis media) | 7.58 | 0.04 | 3.70* | 3.92 |
| Genu varum/vagum | 8.35 | - | -0.79 | -4.02 |
| Spinal deformities (e.g., kyphosis/lordosis) | 2.37 | -0.22** | -6.05** | 0.68 |
| Foramen magnum syndrome/stenosis | - | - | - | - |
| Spinal cord compression/stenosis | 20.17*** | 0.00 | 3.51* | -0.70 |
| Other orthopaedic problems | -11.77* | -0.08 | -2.41 | -1.74 |
| Infections/infestations | -1.68 | -0.21*** | 3.35 | 6.70 |
| Gastroesophageal issues | 2.03 | -0.08 | 3.86* | 4.57 |
| Pain (any) | -5.13 | 0.16*** | 3.15 | -9.12 |
| **Constant** | 86.83*** | 0.92*** | 76.64*** | 92.05*** |

The choice to include z-score or absolute height in regression analyses was based on qualitative assessment of the strength and significance of the estimated correlation coefficients between the two variables and each modelled outcome, respectively. Height z-scores (age- and gender-matched) were computed using World Health Organisation growth tables by gender [de Onis et al., 2007 (22)]. Complications were included as the incidence proportion (within five years of the index date) of all examined complications.

*** p<0.01, ** p<0.05, * p<0.1

**Abbreviations:** PedsQL: pediatric quality of life inventory; QoL: quality of life; QoLISSY: Quality of Life in Short Stature Youth; WeeFIM: paediatric Functional Independence Measure; SDS: standard deviations.
